# Quantum architecture search via truly proximal policy optimization

**DOI:** 10.1038/s41598-023-32349-2

**Published:** 2023-03-29

**Authors:** Xianchao Zhu, Xiaokai Hou

**Affiliations:** 1 School of Artificial Intelligence and Big Data, Henan University of Technology, Zhengzhou, 450001 China; 2 Institute of Fundamental and Frontier Sciences, University of Electronic Science and Technology of China, Chengdu, China

**Keywords:** Computer science, Computational science

## Abstract

Quantum Architecture Search (QAS) is a process of voluntarily designing quantum circuit architectures using intelligent algorithms. Recently, Kuo et al. (Quantum architecture search via deep reinforcement learning. arXiv preprint arXiv:2104.07715, 2021) proposed a deep reinforcement learning-based QAS (QAS-PPO) method, which used the Proximal Policy Optimization (PPO) algorithm to automatically generate the quantum circuit without any expert knowledge in physics. However, QAS-PPO can neither strictly limit the probability ratio between old and new policies nor enforce well-defined trust domain constraints, resulting in poor performance. In this paper, we present a new deep reinforcement learning-based QAS method, called Trust Region-based PPO with Rollback for QAS (QAS-TR-PPO-RB), to automatically build the quantum gates sequence from the density matrix only. Specifically, inspired by the research work of Wang, we employ an improved clipping function to implement the rollback behavior to limit the probability ratio between the new strategy and the old strategy. In addition, we use the triggering condition of the clipping based on the trust domain to optimize the policy by restricting the policy within the trust domain, which leads to guaranteed monotone improvement. Experiments on several multi-qubit circuits demonstrate that our presented method achieves better policy performance and lower algorithm running time than the original deep reinforcement learning-based QAS method.

## Introduction

Reinforcement learning (RL)^1^ has achieved great success and demonstrated human or superhuman abilities in various tasks, such as mastering video games^2–5^ and the game of Go^6, 7^. With such success, it is natural to apply such technologies to scientific fields that require complex control capabilities. In fact, RL has been used to study quantum control^8–14^, quantum error correction^15–18^ and the optimization of variational quantum algorithms^19–22^.

RL has also been used to optimize the structure and parameters of neural networks, which is called Neural Architecture Search (NAS)^23^. Specifically, NAS trains an RL agent to sequentially add different neural networks components (such as convolution operation, residual connection, and pooling) and then automatically generates a high-performance neural network by evaluating the model’s performance to adjust these components structure. NAS is already comparable to human experts in specific tasks, effectively reducing neural networks’ use and implementation costs^24–31^.

Quantum algorithms are proven to have exponential or quadratic operational efficiency improvements in solving specific problems compared to classical algorithms^32, 33^, such as integer factorization^34^ and unstructured database searches^35^. Recent studies in variational quantum algorithms (VQA) have applied quantum computing to many scientific domains, including molecular dynamical studies^36^, quantum optimization^37, 38^ and various quantum machine learning (QML) applications such as regression^39–41^, classification^40, 42–56^, generative modeling^57–62^, deep reinforcement learning^63–69^, sequence modeling^39, 70, 71^, speech identification^72^, distance metric learning^73, 74^, transfer learning^46^ and federated learning^75^. However, designing a quantum circuit to solve a specific task is not easy because it requires domain knowledge and sometimes extraordinary insight.

Recently, a deep reinforcement learning-based Quantum Architecture Search (QAS-PPO) approach is proposed to automatically generate the quantum circuit via the Proximal Policy Optimization (PPO) algorithm without any expert knowledge in physics^76^. Specifically, QAS-PPO uses the PPO^77^ method to optimize the interaction of the RL agent with a quantum simulator to learn the target quantum state. During the interaction, the agent sequentially generates an output action as a candidate for a quantum gate or quantum operation placed on the circuit. Then the fidelity of generated quantum circuit is evaluated to determine the agent whether the agent has reached the goal. This process is performed iteratively to train the RL agent. Despite its success, QAS-PPO can neither strictly limit the probability ratio between old and new policies nor enforce well-defined trust domain constraints, resulting in poor performance.

This paper proposes a new deep reinforcement learning-based QAS approach, named Trust Region-based PPO with Rollback for QAS (QAS-TR-PPO-RB), to automatically build the quantum gates sequence from the density matrix only. Specifically, inspired by the research work of Wang et al.^78^, we adopt an improved clipping function to implement the rollback behavior to limit the probability ratio between the new strategy and the old strategy to prevent the strategy from being pushed away during training. Moreover, we optimize the strategy within the trust region by replacing the clipped trigger conditions with those based on the trust region to guarantee monotonic improvement. Experimental results on several benchmark tasks demonstrate that the proposed method observably improves policy performance and algorithm running time compared to the original deep reinforcement learning-based QAS methods.

The rest of this paper is arranged as follows. “Preliminaries” presents preliminaries on reinforcement learning, advantage actor-critic (A2C), proximal policy optimization (PPO) and quantum architecture search. The QAS-PPO method is reviewed in “Quantum architecture search with deep reinforcement learning”. “Methods” proposes a new deep reinforcement learning-based QAS algorithm, called Trust Region-based PPO with Rollback for QAS (QAS-TR-PPO-RB). Specifically, we adopt an improved clipping function to implement the rollback behavior to limit the probability ratio between the new strategy and the old strategy to prevent the strategy from being pushed away during training. In “Experiments”, we present several experimental comparative results for the automatic generation of quantum circuits for multi-qubit target states to show the superiority of our presented method. Finally, we conclude this paper in “Conclusion”.

## Preliminaries

### Reinforcement learning

The reinforcement learning (RL) algorithm that maximizes the value function is called value-based reinforcement learning. Unlike value-based RL, which learns a value function and uses it as a reference to generate decisions at each step, another RL method is called policy gradient. In this method, the strategy function $\pi (a|s;\theta )$ is parameterized with the parameters $\theta$. Then $\theta$ will be affected by the optimization procedure, which rises gradient ascent on the expected total return ${\mathbb {E}}[R_t]$. One of the classic examples of strategy gradient algorithm is the REINFORCE algorithm^79^. In the standard REINFORCE algorithm, the parameters $\theta$ is updated along the direction $\nabla _{\theta } \log \pi (a_t|s_t;\theta )R_t$, which is the unbiased estimate of $\nabla _{\theta } {\mathbb {E}}[R_t]$. However, the strategy gradient method is affected by the variance of the $\nabla _{\theta } {\mathbb {E}}[R_t]$, making the training very difficult. To reduce the estimate variance and keep it unbiased, the learning function of the state $b_t(s_t)$, which is the baseline, can be substracted from the return value. So the result is $\nabla _{\theta } \log \pi (a_t|s_t;\theta )(R_t-b_t(s_t))$.

### Advantage actor-critic (A2C)

The estimation of the value function is a common choice for the baseline $b_t(s_t) \approx V^{\pi }(s_t)$. This choice usually results in a much lower variance estimation of the strategy gradient. When using the approximation value function as the basic line, the quantity $R_t-b_t=Q(s_t, a_t)-V(s_t)$ can be regarded as the advantage function $A(s_t, a_t)$ of the action in the state $s_t$. Intuitively, one can see this advantage as how nice or nasty the action is compared to the average value in this state $V(s_t)$. For example, if the $Q(s_t, a_t)$ equals to 10 at a given time-step *t*, it is not clear whether $a_t$ is a good action or not. However, if we also know that the $V(s_t)$ equals to, say 2 here, we will imply that $a_t$ may not be bad. Conversely, if the $V(s_t)$ equals to 15, then the advantage is $-5$, meaning that the *Q* value for this action at is well below the average $V(s_t)$ and therefore that action is not good. This approach is called advantage actor-critic (A2C) approach where the strategy $\pi$ is the actor and the value function *V* is the critic ^1^.

### Proximal policy optimization (PPO)

In the strategy gradients method, the policy is optimized by gradient descent according to the policy loss function $L_{policy}({\theta })={\mathbb {E}}_t[-\log \pi (a_t|s_t;\theta )]$. However, the training itself may suffer from instabilities. If the step size of policy update is too small, the training process will be too slow. On the other hand, if the step size is too larger, the training will have a high variance. Proximal policy optimization (PPO) solves this problem by restricting the strategy update step size at each training step^77^. Specifically, The PPO introduces a loss function called the clipped proxy loss function that will restrict the strategy change a small range with the help of a clip. Consider the ratio between the probability of action under present strategy and the probability under anterior strategy $q_t(\theta )=\frac{\pi (a_t|s_t;\theta )}{\pi (a_t|s_t;\theta _{old})}$. If $q_t(\theta )>1$, it means the action is with higher probability in the present strategy than in the old one. And if $0<q_t(\theta )<1$, it means that the action is less probable in the present strategy than in the old one. The new loss function can then be defined as $L_{policy}(\theta ) = {\mathbb {E}}_t[q_t(\theta )A_t]={\mathbb {E}}_t[\frac{\pi (a_t|s_t;\theta )}{\pi (a_t|s_t;\theta _{old})}A_t]$, where $A_t=R_t-V(s_t;\theta )$ is the advantage function. However, if the action under current policy is much more probable than in the previous policy, the ratio $q_t$ may be large, leading to a large policy update step. To circumvent this problem, the original PPO algorithm adds a constraint on the ratio, which can only be in the range 0.8 to 1.2. The modified loss function is defined as follow:

1 $$\begin{aligned} L_{policy}({\theta }) = {\mathbb {E}}_t[-\min (q_t(\theta )A_t,{\mathcal {F}}^{CLIP}(q_t(\theta ),\epsilon )A_t)]. \end{aligned}$$

The clipping function ${\mathcal {F}}^{CLIP}$ is denoted as

2 $$\begin{aligned} {\mathcal {F}}^{CLIP}(q_t(\theta ),\epsilon )= {\left\{ \begin{array}{ll} 1-\epsilon , &{}\text{ if } \; q_t(\theta ) \le 1-\epsilon \\ 1+\epsilon , &{}\text{ if } \; q_t(\theta ) \ge 1+\epsilon \\ q_t(\theta ) &{}\text{ else }, \end{array}\right. }, \end{aligned}$$

where the $(1-\epsilon ,1+\epsilon )$ represents the clipping range, $\epsilon \in [0,1]$ is the clip hyperparameter (common choice is 0.2).

Finally, the value loss and entropy bonus are added into the total loss function as usual: $L(\theta ) = L_{policy}({\theta })+c_1L_{value}({\theta })-c_2H({\theta })$ where $$H({\theta })={\mathbb {E}}_t[H_t]({\theta })={\mathbb {E}}_t[-\begin{matrix} \sum _{j} \pi (a_j|s_t;\theta ) \log (\pi (a_j|s_t;\theta )) \end{matrix}]$$ is the entropy bonus which is used to encourage exploration and $L_{value}({\theta })={\mathbb {E}}_t[\Vert R_t-V(s_t;\theta )\Vert ^2]$ is the value loss.

### Quantum architecture search

Quantum architecture search (QAS) is a class of approaches using algorithms such as quantum simulated annealing (QSA)^80, 81^, quantum evolutionary algorithm (QEA)^82, 83^, quantum machine learning (QML)^84–87^, and quantum reinforcement learning (QRL)^76, 88–90^ intelligent algorithms to voluntarily search for the best quantum circuit for a given target quantum state. Existing research work shows that quantum circuits generated by QAS methods based on variational quantum algorithms have reached or even surpassed quantum circuits designed based on human expertise. However, when such quantum architecture search algorithms automatically generate quantum circuits in discrete environments, they often need to evaluate the performance of many quantum circuits with different structures, resulting in colossal resource consumption. Recently, Zhang et al. presented a Differentiable Quantum Architecture Search (DQAS) method, which expanded the space to be searched from discrete domain to continuous domain and used gradient descent to optimize the entire quantum circuit generation process to achieve relatively high performance^91^.

## Quantum architecture search with deep reinforcement learning

Given the original quantum state ${|{0...0}\rangle }$ and the target quantum state, the goal is to produce a quantum circuit that converts the original state into the target state within a specific fidelity threshold. En-Jui Kuo et al. use the Pauli measurement as an observation, which is a often-used setting for quantum mechanics. Then they adopt two RL algorithms (PPO and A2C) respectively to achieve the above goal^76^. Specifically, environment *E* represents a quantum computer or quantum simulator. The RL agent is hosted on a classic computer and interacts with environment *E*. At each iteration step, the RL agent selects an action *a* from the set of possible actions *A*, consisting of different quantum operations. After the RL agent updates the quantum circuit based on the selected action, environment *E* tests the newly generated circuit and computes the fidelity between the given target quantum state and the currently developed state quantum state. If the calculated fidelity has reached or exceeded a predefined threshold, the round ends, and the RL agent will receive a positive feedback reward. Elsewise, the RL agent will receive a negative feedback reward. This process continues until the maximum number of steps required for the iteration will terminate. The optimization of the algorithm in this interaction can be realized by using reinforcement learning algorithm A2C or PPO.

Given the number of qubits $n\in N$, the initial quantum state ${|{0}\rangle }^{\otimes ^n}$, the target state, the tolerance error, and a set of quantum gates ${\mathbb {G}}$, the goal of the algorithm is to discover a quantum circuit ${\mathcal {C}}$ by construsting an objective function ${\mathcal {F}}$:

3 $$\begin{aligned} {\mathcal {F}}:({|{0}\rangle }^{\otimes ^n},{|{\psi }\rangle },\epsilon ,{\mathbb {G}}) \rightarrow {\mathcal {C}} \end{aligned}$$

such that $1 \ge D({|{\psi }\rangle },{\mathcal {C}}({|{0}\rangle }^{\otimes ^n})) \ge 1-\epsilon$, where ${\mathcal {C}}$ is composed of gates $g \in {\mathbb {G}}$ and *D* is a distance metric between two quantum states (larger is better). In this paper, we use the fidelity^92^ as our distance *D*. Given two density operators $\rho$ and $\sigma$, the fidelity between two operators is usually expressed as $F(\rho ,\sigma )=[tr \sqrt{\sqrt{\rho }\sigma \sqrt{\rho }}]^2$. In particular, in the case where $\rho$ and $\sigma$ represent pure quantum states, i.e., $\rho ={|{\phi _{\rho }}\rangle }{\langle {\phi _{\rho }}|}$ and $\sigma ={|{\phi _{\sigma }}\rangle }{\langle {\phi _{\sigma }}|}$, respectively, the original expression can be reduced to the inner product of the two quantum states: $F(\rho ,\sigma )=|{\langle {\phi _{\rho }|\phi _{\sigma }}\rangle }|^2$.

Furthermore, En-Jui Kuo et al. verified the performance of their proposed deep reinforcement learning-based QAS algorithm using Bell states and Greenberg–Horn–Zehlinger (GHZ) states as target quantum states, respectively.

A Bell state achieves maximal two-qubit entanglement,

4 $$\begin{aligned} {|{Bell}\rangle }=\frac{1}{\sqrt{2}}({|{00}\rangle }+{|{11}\rangle }). \end{aligned}$$

To generate a Bell state, En-Jui Kuo et al. picked the observation to be the expectation values of Pauli matrices on each qubits $\{ {\langle {\sigma _{j}^i}\rangle }|i\in {0,1}, j\in {x,y,z}\}$. The action set ${\mathbb {G}}$ is

5 $$\begin{aligned} {\mathbb {G}}=\bigcup _0^{n-1} {U_i(\frac{\pi }{4}),X_i,Y_i,Z_i,H_i,CNOT_{i,(i+1)(mod2)}}, \end{aligned}$$

where $n=2$ (for two qubits), $$U_i(\theta )= \begin{pmatrix} 1&{}0 \\ 0&{}exp(i\theta ) \end{pmatrix}$$ is the single-qubit rotation applied to the *i*-th qubit around the *Z*-axis, $X_i \equiv \sigma ^i_x$ denotes the Pauli-*X* gate and likewise for $Y_i$ and $Z_i$, $H_i$ represents the Hadamard gate, and $CNOT_{i,j}$ is the CNOT gate where the *i*-th qubit is the control bit, and the *j*-th qubit is the target bit, so there are 12 actions in total.

A GHZ state is a multi-qubit generalization of the Bell state, in which an equal superposition between the lowest and the highest energy states is created.

6 $$\begin{aligned} {|{GHZ}\rangle }=\frac{1}{\sqrt{2}}({|{000}\rangle }+{|{111}\rangle }). \end{aligned}$$

To generate the 3-qubit GHZ state, En-Jui Kuo et al. adopted the expectation values of individual qubit’s Pauli matrices, resuting in 9 observables in the aggregate. For the actions, En-Jui Kuo et al. selected the same single-qubit gates as in Eq. (6), and sixe *CNOT* gate as two-qubit gates.

Despite its success, QAS-PPO can neither strictly limit the probability ratio between old and new policies nor enforce well-defined trust domain constraints, resulting in poor performance. The former problem is mainly due to the inability of PPO to eliminate the incentives of pushing away the strategy, while the latter situation is primarily due to the essential difference of the two types of constraints used by PPO and Trust Region Policy Optimization (TRPO), respectively.

## Methods

In this section, to address above issue, we propose a new deep reinforcement learning-based QAS approach, called Trust Region-based PPO with Rollback for QAS (QAS-TR-PPO-RB). More realistically adhering to the “proximal” property-bound strategy within the trust region, our method can significantly improve over original deep reinforcement learning-based QAS approaches in terms of policy performance and sample efficiency.

### Analysis of the “proximal” property of PPO

PPO limits the strategy by reducing the probability ratio between old and new policies. However, in practice, the known probability ratio is not limited to the clipping range. A significant factor in this problem is that the limiting mechanism cannot eliminate the excitation from the overall target function $L(\theta )$, pushing this out-of-the-range $q_t(\theta )$ further beyond the limit^93^. Moreover, PPO does not explicitly impose a trust domain constraint on the probability ratio between old and new policies, i.e., the *KL*-divergence between the two strategies. Even if the probability ratio $q_t(\theta )$ is bounded, the corresponding *KL*-divergence $D_{KL}^{s_t}(\theta _{old},\theta )$ is not necessarily bounded^78^.

### PPO with rollback for quantum architecture search

As mentioned in “Analysis of the “proximal” property of PPO”, the PPO method used in the QAS-PPO method cannot strictly constrain the range of probability ratio: the limiting mechanism cannot eliminate the motivation to drive $q_t(\theta )$ beyond the limiting range, in fact, $q_t(\theta )$ often deviates from the constraints of this mechanism ultimately lead to poor performance. We solve this problem by replacing the clip function ${\mathcal {F}}^{CLIP}$ with a rollback function whose mathematical expression follows.

7 $$\begin{aligned} {\mathcal {F}}^{RB}(q_t(\theta ),\epsilon , \alpha )= {\left\{ \begin{array}{ll} -\alpha q_t(\theta )+ (1+\alpha ) (1-\epsilon ), &{}\text{ if } \; q_t(\theta ) \le 1-\epsilon \\ -\alpha q_t(\theta )+ (1+\alpha ) (1+\epsilon ), &{}\text{ if } \; q_t(\theta ) \ge 1+\epsilon \\ q_t(\theta ) &{}\text{ otherwise }, \end{array}\right. } \end{aligned}$$

where $\alpha$ represents the hyper-parameter that controls the intensity of the rollback. The new overall target function is $L^{RB}(\theta )$. When $q_t(\theta )$ exceeds the limit range, the rollback function ${\mathcal {F}}^{RB}(q_t(\theta ),\epsilon , \alpha )$ will produce passive stimulation. Therefore, it can offset the excitation from the overall target function $L^{RB}(\theta )$ to a certain extent. The rollback operation prevents the probability ratio $q_t(\theta )$ from being squeezed out more strongly than the original clip function^78^.

The pseudocode for the PPO with Rollback for Quantum Architecture Search approach is shown below:
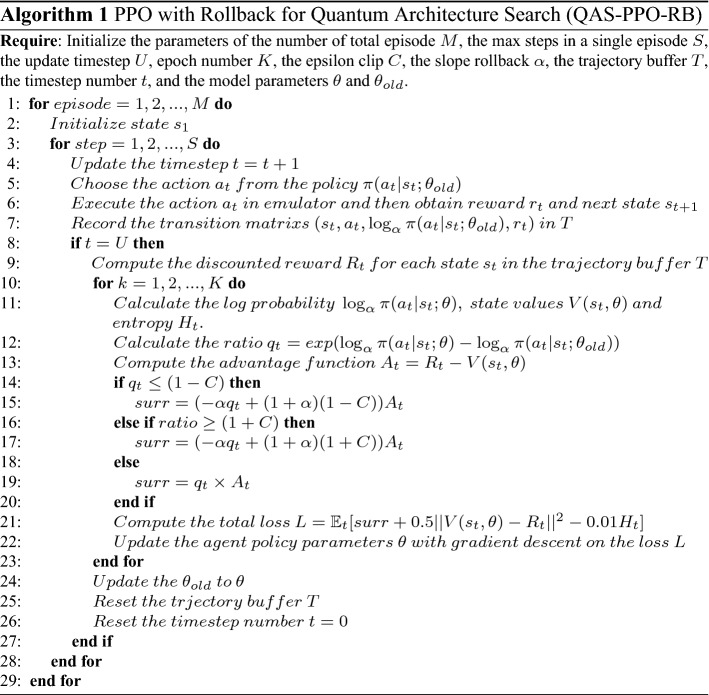


### Trust region-based PPO for quantum architecture search

As mentioned in “Analysis of the “proximal” property of PPO”, the clipping function in the original deep reinforcement learning-based QAS method uses the probability ratio as the element of the clipping trigger condition, which makes the difference between the ratio-based constraints and the trust domain-based constraints used: the constraint probability ratio is not enough to constrain the *KL*-divergence, which ultimately leads to poor performance. Therefore, we replace the ratio-based clipping function with a trust domain-based clipping function. Formally, when the strategy $\pi _{\theta }$ exceeds the trust domain, the probability ratio is tailored,

8 $$\begin{aligned} {\mathcal {F}}^{TR}(q_t(\theta ),\delta )= {\left\{ \begin{array}{ll} q_t(\theta _{old}), &{}\text{ if } \; D_{KL}^{s_t}(\theta _{old},\theta ) \ge \delta \\ q_t(\theta ) &{}\text{ else }, \end{array}\right. } \end{aligned}$$

where $\delta$ is the hyperparameter, $q_t(\theta _{old})=1$ is a constant. When the strategy $\pi _{\theta }$ exceeds the trust region, that is, $D_{KL}^{s_t}(\theta _{old},\theta ) \ge \delta$, the incentive for updating the strategy is removed. Although the clipped value $q_t(\theta _{old})$ may make the proxy target function uncontinuous, this discontinuity will not influence the optimization of the parameter $\theta$ because the gradient will not be affected by the constant value.

In general, our proposed QAS-TR-PPO method combines the advantages of TRPO and PPO: it is theoretically reasonable (subject to the trust domain), is easy to implement, and only need to do one-rank optimization. On the one hand, our approach does not require *KL*-divergence $D_{KL}^{s_t}(\theta _{old},\theta )$ to optimize $\theta$. $D_{KL}^{s_t}(\theta _{old},\theta )$ calculates to determine whether to $q_t(\theta )$ or not. Compared with the PPO used in the original method, our method uses a different strategy metric to limit the strategy. Specifically, unlike PPO, the ratio-based metric $\frac{\pi (a_t|s_t;\theta )}{\pi (a_t|s_t;\theta _{old})}$ is used to impose element-by-element constraints on the sampling action points. Our method uses a trusted domain the *KL*-divergence $$\begin{matrix} \sum _{a} \pi (a_t|s_t;\theta _{old}) \log \frac{\pi (a_t|s_t;\theta )}{\pi (a_t|s_t;\theta _{old})} \end{matrix}$$ based on the trust region to impose a summation constraint on the action space. Crucially, the central willingness is that ratio-based regulations can impose relatively strict restrictions on actions that the old strategy does not like, that is, $\pi _{\theta _{{old}}}$ is small, which may result in finite sample efficiency when the strategy is initialized from a wrong solution. On the contrary, the trust domain-based approach we adopted has no such prejudice and tends to show higher sample efficiency in reality^78^.

Finally, we should pay attention to the importance of the $\min (\cdot ,\cdot )$ operation for all variants of PPO. The operation function $\min (\cdot ,\cdot )$ is denoted as:

9 $$\begin{aligned} L^{TR}_{policy}(\theta ) = {\mathbb {E}}_t[-\min (q_t(\theta )A_t,{\mathcal {F}}^{TR}(q_t(\theta ),\delta )A_t)]. \end{aligned}$$

Schulman et al. proposed that this additional $\min (\cdot ,\cdot )$ operation made $L^{TR}_{policy}{\theta }$ become a lower bound on the unclipped target function $q_t(\theta )A_t$^94^. As Eq. (11) expresses, there is no target of $\min (\cdot ,\cdot )$ operation, namely ${\mathcal {F}}^{TR}(q_t(\theta ),\delta )A_t$. Once the policy violates the trust region, it will stop updating, even if the target value is less than the original value, that is, $q_t(\theta )A_t \le q_t(\theta _{old})A_t$. The $\min (\cdot ,\cdot )$ operation virtually provides a remedy for this problem. Therefore, Eq. (12) can be rewritten as

10 $$\begin{aligned} L^{TR}_{policy}(\theta )= {\left\{ \begin{array}{ll} q_t(\theta _{old})A_t, &{}\text{ if } \; D_{KL}^{s_t}(\theta _{old},\theta ) \ge \delta \, and \, q_t(\theta )A_t \ge q_t(\theta _{old})A_t \\ q_t(\theta )A_t &{}\text{ else }. \end{array}\right. } \end{aligned}$$

In this way, the ratio will be clipped only when the target value increases (and the policy violates the constraints). The Trust Region-based PPO for Quantum Architecture Search method is as follows (as shown in Algorithm 2):
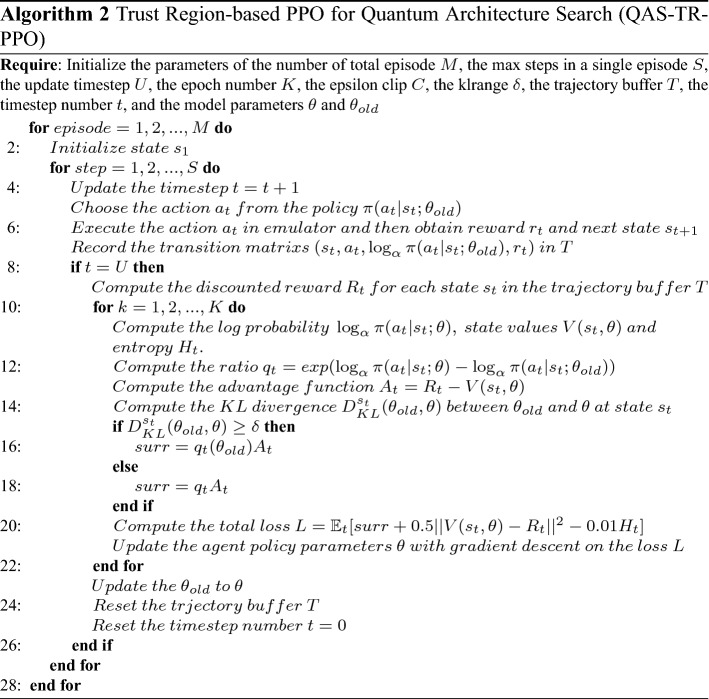


### Trust region-based PPO with rollback for quantum architecture search

However, the tailoring based on the trust domain may still have the problem of an unbounded probability ratio. When the strategy exceeds the trust region, the method proposed above does not provide any negative incentives, leading to poor performance. Therefore, we solve this problem by combining the tailoring based on the trust domain and the rollback mechanism.

11 $$\begin{aligned} {\mathcal {F}}^{TR-RB}(q_t(\theta ),\delta , \alpha )= {\left\{ \begin{array}{ll} -\alpha q_t(\theta _{old}), &{}\text{ if } \; D_{KL}^{s_t}(\theta _{old},\theta ) \ge \delta \\ q_t(\theta ) &{}\text{ else }. \end{array}\right. } \end{aligned}$$

As shown in Eq. (13), when $\pi _{\theta }$ exceeds the trust domain, our proposed ${\mathcal {F}}^{TR-RB}(q_t(\theta ),\delta , \alpha )$ method will produce negative excitation. Trust Region-based PPO with Rollback for Quantum Architecture Search method is as follows (as shown in Algorithm 3):
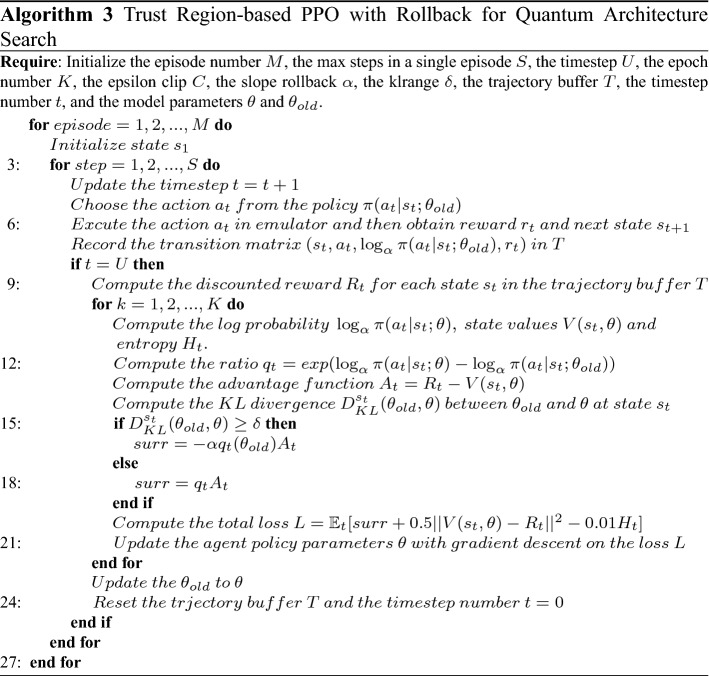


## Experiments

### Experimental settings

#### Optimizer

In this paper, we employ the Adam optimizer for training the RL agent in the A2C, PPO, PPO-RB, TR-PPO and TR-PPO-RB cases^95–99^. Adam is one of the gradient-descent methods which calculates the self-adaptive learning rates of each parameter. Furthermore, Adam stores both the exponentially damping mean of gradient $g_t$ and its square $g^2_t$,

12 $$\begin{aligned} \mu _t= & {} \zeta _1 \mu _{t-1}+(1-\zeta _1)g_t, \end{aligned}$$

13 $$\begin{aligned} \nu _t= & {} \zeta _2 \nu _{t-1}+(1-\zeta _2)g^2_t, \end{aligned}$$

where $\zeta _1$ and $\zeta _2$ are hyperparameters. We use $\zeta _1=0.9$ and $\zeta _2=0.999$ in this papaer. The $\mu _t$ and $v_t$ are adjustable according to the following equation to offset the biases towards 0,

14 $$\begin{aligned} {\hat{\mu }}_t= & {} \frac{m_t}{1-\zeta ^t_1}, \end{aligned}$$

15 $$\begin{aligned} {\hat{\nu }}_t= & {} \frac{\nu _t}{1-\zeta ^t_2}. \end{aligned}$$

The parameters $\theta _t$ in the our method in the time step *t* are then updated according to the following equation,

16 $$\begin{aligned} \theta _{t+1}=\theta _t-\frac{\eta }{\sqrt{{\hat{\nu }}_t}+\epsilon }{\hat{\mu }}_t. \end{aligned}$$

#### Quantum noise in quantum simulator

In this paper, we consider two forms of errors: gate errors and measurement errors^100^. The gate error refers to the defect in any quantum operation during the algorithm’s execution, and the measurement error refers to the error generated in the quantum measurement process. Specifically, for gate error, we consider the depolarizing noise, which replaces the state of any qubit with a stochastic state of probability $p_{gate}$. For the measurement error, we think about a stochastic flip between 0 and 1 with probability $p_{meas}$ immediately before the actual measurement. We use the following noise configuration in the simulation software to test the manifestation of the agent of our proposed approach:

- error rate (both $p_{gate}$ and $p_{meas}$)= 0.001
- error rate (both $p_{gate}$ and $p_{meas}$)= 0.005

#### Density matrix of quantum states

The generic form of a density matrix $\rho$ of a quantum state under the basis ${|{\psi _j}\rangle }$ is,

17 $$\begin{aligned} \rho =\sum _{j} p_j {|{\psi _j}\rangle }{\langle {\psi _j}|}, \end{aligned}$$

where $p_j$ denotes the probability that the quantum system is in the pure state ${|{\psi _j}\rangle }$ such that $$\begin{matrix} \sum _{j} p_j=1 \end{matrix}$$. For example, the density matrix of the Bell state adopted in this paper is ${|{Bell}\rangle }=\frac{1}{\sqrt{2}}({|{00}\rangle }+{|{11}\rangle })$. Its corresponding density matrix $\rho$ is then given by

18 $$\begin{aligned} {|{Bell}\rangle }{\langle {Bell}|}=\frac{1}{2}({|{00}\rangle }{\langle {00}|}+{|{00}\rangle }{\langle {11}|}+{|{11}\rangle }{\langle {00}|}+{|{11}\rangle }{\langle {11}|}). \end{aligned}$$

#### Quantum state tomography

Quantum state tomography (QST), which reconstructs quantum states of quantum systems through quantum measurements, plays an important role in verifying and benchmarking quantum devices in various quantum information processing tasks. Expand the density matrix in the Pauli basis of *N* qubits,

19 $$\begin{aligned} \rho =\frac{1}{2^N}\sum _{i_1, \ldots , i_{N}=0}^3 C_{i_1,\ldots , i_{N}} \sigma _{i_1}\otimes \cdots \otimes \sigma _{i_N}, \end{aligned}$$

where $4^N-1$ measurement operations are required to determine $\rho$ (minus one due to the conservation of probability, $Tr(\rho )=1$). More generally, the measurement using $4^N-1$ linearly independent projection operators can uniquely determine the density matrix, where Eq. (14) is a special case with the projectors being the Pauli operators. Therefore, the number of measurements increase exponentially in the qubit number *N*, which poses a huge challenge for verifying multi-qubit quantum states in any experiments, and under a limited number of shots, the expectation values of ${\rho _{i_1, \ldots , i_{N}}}$ can only be measured within certain accuracy. In this paper, we adopt IBM’s Qiskit software package to perform the quantum state tomography simulations^100^.

#### Customized OpenAI gym environment

We use a customized OpenAI gym environment^101^ to verify the performance of our proposed algorithm. In this experimental environment, the objective quantum state, the fidelity threshold, and the quantum computation backend (real machine or simulator software) are set by the user in the form of parameters. In addition, users can also customize the noise mode. Specifically, we use the following parameter settings to build the test environment:

- Observation: The agent receives Pauli-*X*, *Y*, *Z* expected values on each qubit.
- Action: The RL agent will choose a quantum gate that runs on a specific qubit.
- Reward: Before successfully reaching the goal, the agent will receive a $-0.01$ reward for every step to encourage exploring the shortest path. When the agent reaches the goal, it will obtain a reward of *F*.

#### Parameter settings

In this paper, we think about five RL algorithms in this paper, their parameter setting are shown as follow:

- A2C: learning rate $\eta =10^{-4}$, discount factor $\gamma =0.99$.
- PPO: $\eta =0.002$, $\gamma =0.99$, clip range parameter $C=0.2$, update epoch number $K=4$.
- PPO-RB: $\eta =0.002$, $\gamma =0.99$, clip range parameter $C=0.2$, update epoch number $K=4$, slope rollback $\alpha =-0.3$.
- TR-PPO: $\eta =0.002$, $\gamma =0.99$, clip range parameter $C=0.2$, update epoch number $K=4$, klrange $\delta =0.03$.
- TR-PPO-RB: $\eta =0.002$, $\gamma =0.99$, clip range parameter $C=0.2$, update epoch number $K=4$, klrange $\delta =0.03$, slope rollback $\alpha =-0.3$.

### Noise-free environments performance

#### 2-Qubit Bell state

**Figure 1:**
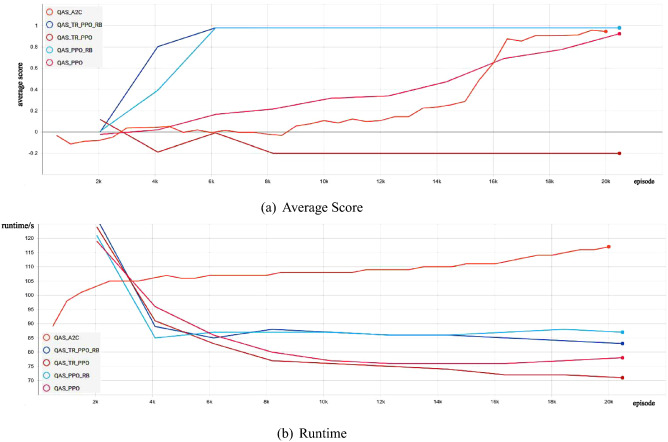
Comparison of the average score and the runtime with different deep reinforcement learning-based QAS methods for Quantum Architecture Search on noise-free Two-Qubit system.

**Figure 2:**
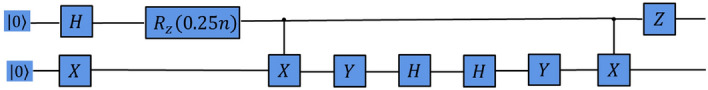
Quantum circuit for the Bell state generated by the RL agent on noise-free Two-Qubit system.

Firstly, we show the experimental comparison results of different deep reinforcement learning-based QAS methods generating the 2-qubit Bell state from scratch in a noise-free environment (as shown in Fig. 1). We can find that these deep reinforcement learning-based QAS methods can successfully train RL agents to synthesize Bell states, however, under the same neural network, our proposed algorithm obtains better policy performance and less running time than other methods. Figure 2 shows the Bell state quantum circuit generated by our proposed method on a noise-free two-qubit system.

#### 3-Qubit GHZ state

**Figure 3:**
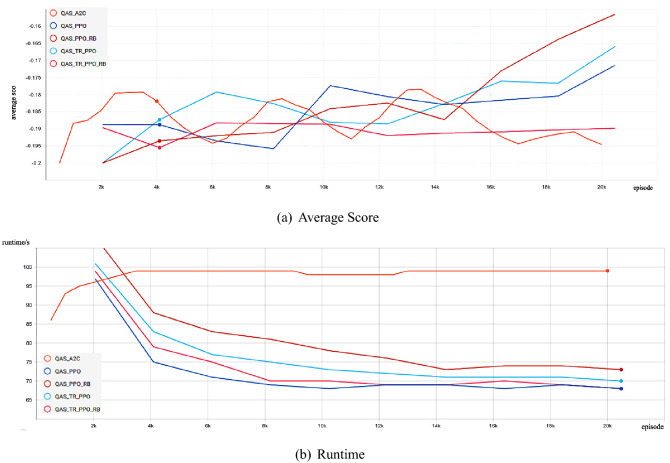
Comparison of the average score and the runtime with different deep reinforcement learning-based QAS methods on noise-free Three-Qubit system.

**Figure 4:**
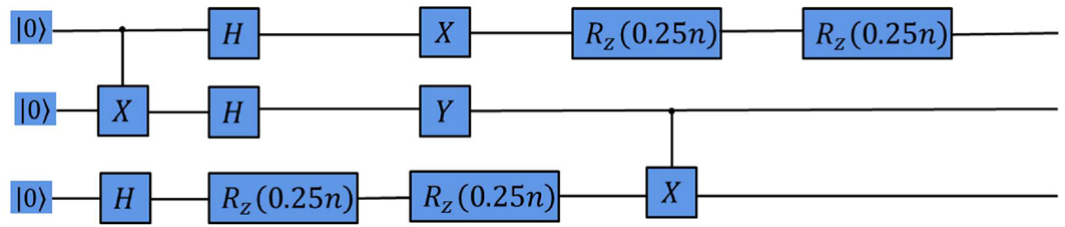
Quantum circuit for the GHZ state generated by the RL agent on noise-free Three-Qubit system.

Secondly, we show the experimental comparison results of different deep reinforcement learning-based QAS methods generating the 3-qubit GHZ state from scratch in a noise-free environment (as shown in Fig. 3). We can find that these deep reinforcement learning-based QAS methods can successfully train RL agents to synthesize GHZ states, however, under the same neural network, our proposed method reaches optimal policy performance faster and the algorithm running time is less compared to other methods. In Fig. 4, we provide the quantum circuit for GHZ state generated by our proposed method on a noise-free three-qubit system.

#### 4-Qubit SK Ising spin glass state

Thirdly, we focus on a classical problem in combination optimization, namely, the SK Ising spin glass with the energy function

20 $$\begin{aligned} C=\frac{1}{\sqrt{n}}\sum _{i,j=1}^n J_{ij} \sigma _i^z \sigma _j^z+\sum _{i=1}^n h_{i} \sigma _i^z, \end{aligned}$$

where $J_{ij}$ and $h_{i}$ represent independent Gaussian stochastic variables with zero-mean and zero-variance $J^2=h^2=1$, and each $\sigma ^z$ spin can take the values $\pm 1$. We use the Metropolis algorithm to calculate the ground state of the Hamiltonian system of the SK Ising Spin Glass model as the target quantum state.

**Figure 5:**
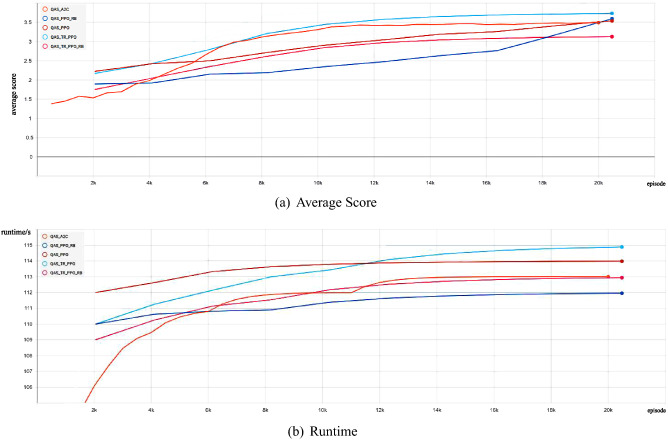
Comparison of the average score and the runtime with different deep reinforcement learning-based QAS methods on noise-free Four-Qubit system.

**Figure 6:**
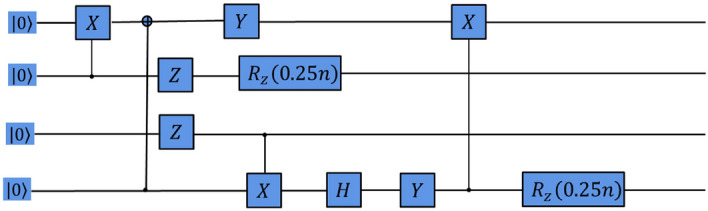
Quantum circuit for the 4-qubit SK Ising spin galss state generated by the RL agent on noise-free Four-Qubit system.

We show the experimental comparison results of different deep reinforcement learning-based QAS methods generating the 4-qubit SK Ising spin galss state from scratch in a noise-free environment (as shown in Fig. 5). We can observe that these deep reinforcement learning-based QAS methods can successfully train RL agents to synthesize SK Ising spin galss states, however, under the same neural network, our proposed algorithm obtains better policy performance and less running time than other methods. Figure 6 shows the SK Ising spin galss state quantum circuit generated by our proposed method on a noise-free four-qubit system.

### Noisy environments performance

#### 2-Qubit Bell state

**Figure 7:**
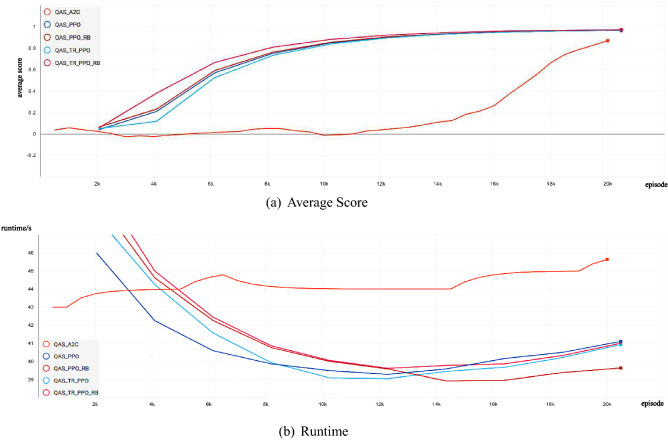
Comparison of the average score and the runtime with different deep reinforcement learning-based QAS methods on noisy Two-Qubit system.

**Figure 8:**
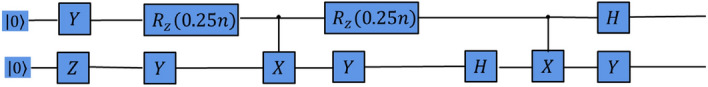
Quantum circuit for the Bell state generated by the RL agent on noisy Two-Qubit system.

Fourthly, we show the experimental comparison results of different deep reinforcement learning-based QAS methods generating the 2-qubit Bell state from scratch in a noisy simulation environment (as shown in Fig. 7). We can observe that these deep reinforcement learning-based QAS methods can successfully train RL agents to synthesize Bell states, however, under the same neural network, our proposed algorithm obtains better policy performance and less running time than other methods. Figure 8 shows the Bell state quantum circuit generated by our proposed method on a noisy simulation two-qubit system.

As shown in Figs. 1 and 7, for the trust region-based clipping methods (QAS-TR-PPO and QAS-TR-PPO-RB), the KL divergences are also smaller than those of QAS-PPO. Especially, QAS-TR-PPO shows the enhanced restriction ability on the KL divergence even it does not incorporate the rollback mechanism. Furthermore, the proportions of out-of-range probability ratios of QAS-TR-PPO-RB are much less than those of the original QAS-PPO during the training process. The probability ratios and the KL divergences of QTR-TR-PPO-RB are also much smaller than those of QAS-PPO. The “rollback” operation on the KL divergence can be regarded as a penalty (regularization) term:

21 $$\begin{aligned} L^{penalty}_{t}(\theta )=q_{t}(\theta )A_{t}-\alpha D_{KL}^s(\theta _{old},\theta ). \end{aligned}$$

The penalty-based methods are usually notorious by the difficulty of adjusting the trade-off coefficient. And PPO-penalty addresses this issue by adaptively adjusting the rollback coefficient $\alpha$ to achieve a target value of the KL divergence. However, the penalty-based PPO does not perform well as the clipping-based one, as it is difficult to find an effective coefficient-adjustig strategy across different tasks. Our method introduces the “clipping” strategy to assisst in restricting policy, i.e., the penalty is enforced only when the policy is out of the trust region. As for when the policy is inside the trust region, the objective function is not affected by the penalty term. Such a mechanism could relieve the difficulty on adjusting the trade-off coefficient, and it will not alter the theoretical property of monotonic improvement (as we will show below). In practice, we found QAS-TR-PPO-RB to be more robust to the coefficient and achieve better performance across different tasks. The clipping technique may be served as an effective method to enforce the restriction, which enjoys low optimization complexity and seems to be more robust.

To analyse the monotonic improvement property, we use the maximum KL divergence instead, i.e.,

22 $$\begin{aligned} L^{TR \_RB}_{policy}(\theta )= q_t(\theta )A_t-{\left\{ \begin{array}{ll} \alpha \max _{s_{t+1} \in S} D_{KL}^{s_{t+1}}(\theta _{old},\theta ), &{}\text{ if } \; \max _{s_{t+1} \in S} D_{KL}^{s_{t+1}}(\theta _{old},\theta ) \ge \delta \, \\ {} &{} and \, q_t(\theta )A_t \ge q_t(\theta _{old})A_t \\ \delta &{}\text{ else }. \end{array}\right. } \end{aligned}$$

in which the maximum KL divergence is also used in TRPO for theoretical analysis. Such objective function also possesses the theoeretical property of the guaranteed monotonic improvement. Let $\theta _{new}^{TR \_RB}=\arg \max _{\theta }L^{TR \_RB}_{policy}(\theta )$ and $\theta _{new}^{TRPO}=\arg \max _{\theta }M(\theta )$ denote the optimal solution of QAS-TR-PPO-RB and TRPO respectively. We have the follow theorem.

##### Theorem 1

*If* $\alpha =C \triangleq max_t \vert A_t \vert 4 \gamma / (1-\gamma )^2$ *and* $\delta \le \max _{s_{t} \in S} D_{KL}^{s_{t}}(\theta _{old},\theta _{new}^{TRPO})$, *then* $\zeta (\theta _{new}^{TR \_RB}) \ge \zeta (\theta _{old})$, *where* $\zeta (\theta )=E_{s^t,a^t}[r(s^t,a^t)]$.

##### Proof

Firstly, we prove two properties of $\theta _{new}^{TRPO}$.

Note that $M(\theta )=E_t[q_t(\theta )A_t]-\alpha \max _{s_{t+1} \in S} D_{KL}^{s_{t+1}}(\theta _{old},\theta )$. As $\theta _{new}^{TRPO}$ is the optimal solution of $M(\theta )$, we have

23 $$\begin{aligned} E_a[q_{s^{t},a} (\theta _{new}^{TRPO})A_{s^{t},a}] \ge E_a[q_{s^{t},a}(\theta _{old})A_{s^{t},a}], \forall s_t \end{aligned}$$

Assume $\theta _{new}$ is an optimal solution of $M(\theta )$ and there exists some $s^{t+1}$ such that $E_a[q_{s^{t+1},a} (\theta ')A_{s^{t+1},a}] \ge E_a[q_{s^{t+1},a}(\theta _{old})A_{s^{t+1},a}]$, then we can construst a new policy

24 $$\begin{aligned} \pi _{\theta ''}(\cdot |s_{t})= {\left\{ \begin{array}{ll} \pi _{\theta _{old}}(\cdot |s_{t}), &{}\text{ if } \; E_a[q_{s^{t+1},a} (\theta ')A_{s^{t+1},a}] \ge E_a[q_{s^{t+1},a}(\theta _{old})A_{s^{t+1},a}]\\ \pi _{\theta '}(\cdot |s_{t}) &{}\text{ else } \end{array}\right. } \end{aligned}$$

We have $M(\pi _{\theta '}) \le M(\pi _{\theta ''})$, which contradicts that $\pi _{\theta '}(\cdot |s_{t})$ is an optimal policy.

Besides, by Eq. (23), we can also obtain that for any $s_t$ there exists at least one $a'$ such that $q_{s^{t},a'} (\theta )A_{s^{t},a'} \ge q_{s^{t},a'}(\theta _{old})A_{s^{t},a'}$. Therefore, by Eq. (22), we have

25 $$\begin{aligned} L^{TR \_RB}_{policy}(\theta _{new}^{TRPO})+\zeta (\theta _{old})=L_{policy}(\theta _{new}^{TRPO})-\alpha \max _{s_{t} \in S} D_{KL}^{s_{t}}(\theta _{old},\theta _{new}^{TRPO}). \end{aligned}$$

Then, we prove that $\theta _{new}^{TRPO}$ is the optimal solution of $L^{TR \_RB}_{policy}$. There are three cases.

- For $\theta '$ which satisfies $\max _{s_{t} \in S} D_{KL}^{s_{t}}(\theta _{old},\theta ') \ge \delta$ and there exist some $a'$ such that $q_{s^{t},a'} (\theta ')A_{s^{t},a'} \ge q_{s^{t},a'}(\theta _{old})A_{s^{t},a'}$ for any $s^t$, we have
  26 $$\begin{aligned} \begin{aligned} L^{TR \_RB}_{policy}(\theta ')+\zeta (\theta _{old}) =L_{policy}(\theta ')-\alpha \max _{s_{t} \in S} D_{KL}^{s_{t}}(\theta _{old},\theta ') \\ \le L_{policy}(\theta _{new}^{TRPO})-\alpha \max _{s_{t} \in S} D_{KL}^{s_{t}}(\theta _{old},\theta _{new}^{TRPO}) \\ =L^{TR \_RB}_{policy}(\theta _{new}^{TRPO})+\zeta (\theta _{old}). \end{aligned} \end{aligned}$$
- For $\theta '$ which satisfies $\max _{s_{t} \in S} D_{KL}^{s_{t}}(\theta _{old},\theta ') \ge \delta$, we have
  27 $$\begin{aligned} \begin{aligned} L^{TR \_RB}_{policy}(\theta ')+\zeta (\theta _{old}) =L_{policy}(\theta ')-\alpha \delta \\ \le L_{policy}(\theta ')-\alpha \max _{s_{t} \in S} D_{KL}^{s_{t}}(\theta _{old},\theta ') \\ \le L_{policy}(\theta _{new}^{TRPO})-\alpha \max _{s_{t} \in S} D_{KL}^{s_{t}}(\theta _{old},\theta _{new}^{TRPO}) \\ =L^{TR \_RB}_{policy}(\theta _{new}^{TRPO})+\zeta (\theta _{old}). \end{aligned} \end{aligned}$$
- We now prove the case of $\theta '$ which satisfies $\max _{s_{t} \in S} D_{KL}^{s_{t}}(\theta _{old},\theta ') \ge \delta$ and there exist some $s^t$ such that $q_{s^{t},a'} (\theta ')A_{s^{t},a'} < q_{s^{t+1},a}(\theta _{old})A_{s^{t+1},a}$ for any *a*, we have
  28 $$\begin{aligned} \begin{aligned} E_a[L^{TR \_RB}_{policy,s^{t},a}(\theta ')] =E_a[q_{s^{t+1},a}(\theta ')]-\alpha \delta \\ < E_a[q_{s^{t+1},a}(\theta _{old})]-\alpha \delta \\ \le E_a[q_{s^{t+1},a}(\theta _{new}^{TRPO})-\alpha \max _{s_{t+1} \in S} D_{KL}^{s_{t+1}}(\theta _{old},\theta _{new}^{TRPO}) \\ =E_a[L^{TR \_RB}_{policy,s^{t+1},a}(\theta _{new}^{TRPO})]. \end{aligned} \end{aligned}$$
  We can construst a new policy
  29 $$\begin{aligned} \pi _{\theta ''}(\cdot |s_{t})= {\left\{ \begin{array}{ll} \pi _{\theta _{new}^{TRPO}}(\cdot |s_{t}), &{}\text{ if } \; s \in \{s^{t+1}\}\\ \pi _{\theta '}(\cdot |s_{t}) &{}\text{ else } \end{array}\right. } \end{aligned}$$
  for which we have
  30 $$\begin{aligned} \begin{aligned} L^{TR \_RB}_{policy}(\theta ')+\zeta (\theta _{old}) =E_{s,a}[L^{TR \_RB}_{policy,s^{t},a}(\theta ')]+\zeta (\theta _{old})\\ <_{s,a}[L^{TR \_RB}_{policy,s^{t},a}(\theta '')]+\zeta (\theta _{old}) \\ =L_{policy}(\theta '')-\alpha \max _{s_{t} \in S} D_{KL}^{s_{t}}(\theta _{old},\theta '') = E_{s,a}[L^{TR \_RB}_{policy,s^{t},a}(\theta '')]+\zeta (\theta _{old})\\ \le M(\theta _{new}^{TRPO}) \\ =L^{TR \_RB}_{policy}(\theta _{new}^{TRPO})+\zeta (\theta _{old}). \end{aligned} \end{aligned}$$

In summary, we have $\zeta (\theta _{new}^{TR \_RB})=\zeta (\theta _{new}^{TRPO})\ge M(\theta _{new}^{TRPO})M(\theta _{old})=\zeta (\theta _{old})$ $\square$

#### 3-Qubit GHZ state

**Figure 9:**
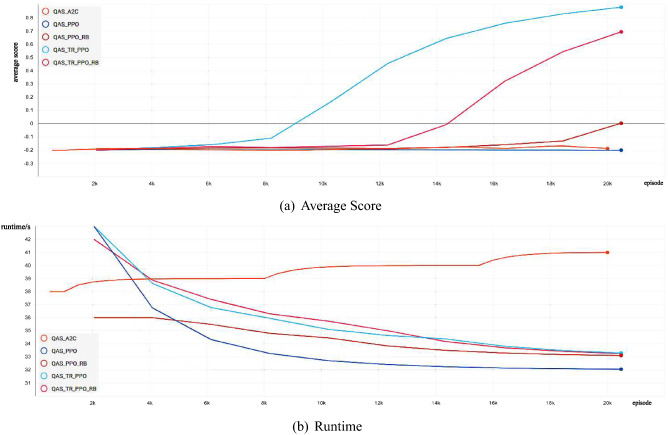
Comparison of the average score and the runtime with different deep reinforcement learning-based QAS methods on noisy Three-Qubit system.

**Figure 10:**
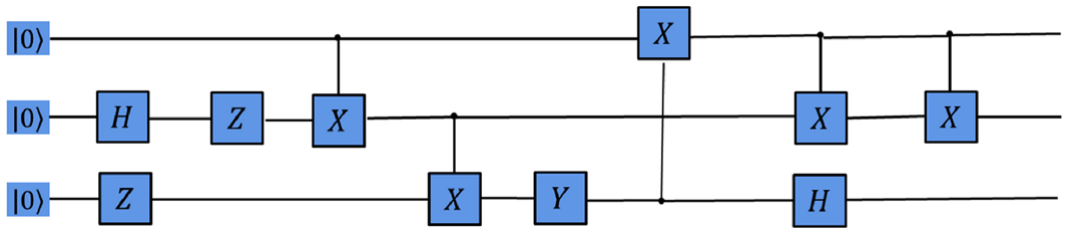
Quantum circuit for the GHZ state generated by the RL agent on noisy Three-Qubit system.

Then, we show the experimental comparison results of different deep reinforcement learning-based QAS methods generating the 3-qubit GHZ state from scratch in a noisy simulation environment. As shown in Fig. 9, we observe that, give the same neural network architeure, our method performs signficantly better than original deep reinforcement learning-based QAS methods in terms of the runtime and the policy performance. Figure 10 shows the GHZ state quantum circuit generated by our proposed method on a noisy simulation three-qubit system.

#### 4-Qubit SK Ising spin glass state

**Figure 11:**
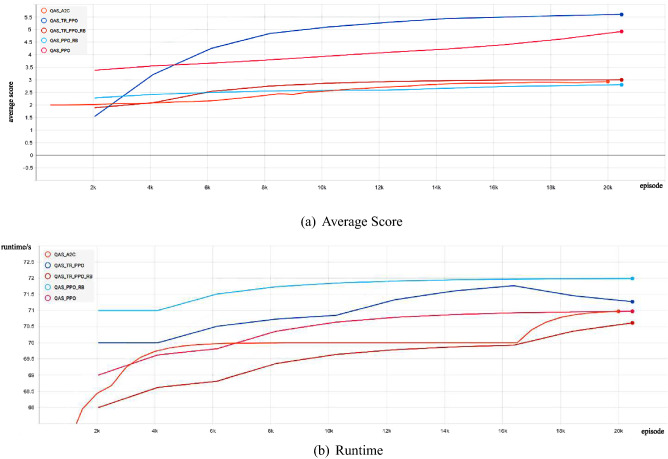
Comparison of the average score and the runtime with different deep reinforcement learning-based QAS methods on noisy Four-Qubit system.

**Figure 12:**
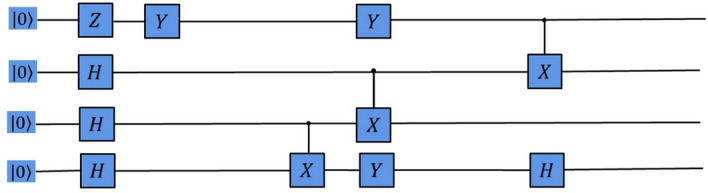
Quantum circuit for the 4-qubit SK Ising spin galss state generated by the RL agent on noisy Four-Qubit system.

Finally, we show the experimental comparison results of different deep reinforcement learning-based QAS methods generating the 4-qubit SK Ising spin galss state from scratch in a noisy environment (as shown in Fig. 11). We can observe that although these deep reinforcement learning-based QAS algorithms can successfully train RL agents to synthesize SK Ising spin galss states, under the same neural network, our proposed approach obtains better policy performance and less running time than other methods. Fig. 12 shows the SK Ising spin galss state quantum circuit generated by our proposed method on a noisy four-qubit system.

## Conclusion

In this paper, we present a new deep reinforcement learning-based QAS approach, named Trust Region-based PPO with Rollback for QAS (QAS-TR-PPO-RB), to automatically build the quantum gates sequence from the density matrix only. Specifically, inspired by the research work of Wang, we adopt an improved clipping function to implement the rollback behavior to limit the probability ratio between the new strategy and the old strategy. Moreover, we optimize the strategy within the trust region by replacing the clipped trigger conditions with those based on the trust region to guarantee monotonic improvement. In this way, our method can improve the original deep reinforcement learning-based QAS methods on policy performance and algorithm running time.

## Data Availability

The datasets used during the current study are available in the Qiskit and Stable-Baselines3 repositories, https://github.com/Qiskit/qiskit and https://stable-baselines3.readthedocs.io/en/master/, respectively.
